# Platelet-Derived Biomarkers: Potential Role in Early Pediatric Serious Bacterial Infection and Sepsis Diagnostics

**DOI:** 10.3390/jcm11216475

**Published:** 2022-10-31

**Authors:** Aiste Pociute, Muhammed Fazil Kottilingal Farook, Algirdas Dagys, Rimantas Kevalas, Goda Laucaityte, Lina Jankauskaite

**Affiliations:** 1 Faculty of Medicine, Lithuanian University of Health Sciences, 44307 Kaunas, Lithuania; 2 Department of Pediatrics, Faculty of Medicine, Lithuanian University of Health Sciences, 44307 Kaunas, Lithuania; 3 Institute of Physiology and Pharmacology, Faculty of Medicine, Lithuanian University of Health Sciences, 44307 Kaunas, Lithuania

**Keywords:** sepsis, SBI, child, platelets, sP-selectin, CXCL7

## Abstract

Fever is the most common complaint of children who are attending a pediatric emergency department (PED). Most of the fever cases are of viral origin; however, the most common markers, such as leucocyte, neutrophil count, or C-reactive protein, are not sensitive or specific enough to distinguish the etiology of fever, especially if children present at the early phase of infection. Currently, platelets have been attributed a role as important sentinels in viral and bacterial infection pathogenesis. Thus, our aim was to analyze different platelet indices, such as PNLR (platelet-to-neutrophil/lymphocyte ratio), PNR (platelet-to-neutrophil ratio) as well as specific secreted proteins, such as sP-selectin, CXCL4, CXCL7, and serotonin. We included 68 children who were referred to PED with the early onset of fever (<12 h). All children with comorbidities, older than five years, and psychiatric diseases, who refused to participate were excluded. All the participants were divided into viral, bacterial, or serious bacterial infection (SBI) groups. All the children underwent blood sampling, and an additional sample was collected for protein analysis. Our analysis revealed statistically significant differences between leucocyte, neutrophil, and CRP levels between SBI and other groups. However, leucocyte and neutrophil counts were within the age norms. A higher PNLR value was observed in a bacterial group, PNR-in viral. As we tested CXCL7 and sP-selectin, alone and together those markers were statistically significant to discriminate SBI and sepsis from other causes of infection. Together with tachypnoe and SpO2 < 94%, it improved the prediction value of sepsis as well as SBI. CXCL4 and serotonin did not differ between the groups. Concluding, CXCL7 and sP-selectin showed promising results in early SBI and sepsis diagnosis.

## 1. Introduction

Fever is the most common complaint of referral to the pediatric emergency department (PED). In most cases, viral or not severe bacterial infection will manifest with fever only, yet, some of the feverish children will have a serious bacterial infection (SBI) including sepsis. Despite introduced vaccination against some of the SBI-inducing pathogens or improved guidelines for an early antibiotic prescription, SBI remains one of the leading causes of children’s mortality worldwide [1]. Fever—one of the diagnostic symptoms of systemic inflammatory response syndrome (SIRS)—is not sensitive nor specific enough to differentiate between viral, bacterial infection, or SBI, especially in the early phase of the infectious process. Together with the clinical features, laboratory tests, such as general blood count (GBC) as well as acute inflammation biomarkers, such as C-reactive protein (CRP) or procalcitonin (PCT), do not improve sensitivity nor specificity to identify the cause of the infection a few hours after the onset of fever [2]. Thus, new, and more reliable, biomarkers are needed to identify the etiological factor in the early phase of infection to prescribe the right treatment as soon as possible [3]. Recently, more data emerged showing platelet involvement in viral and bacterial infection-induced inflammation and host immune response. Activated platelets sense pathogens through specific receptors, resulting in leukocyte, neutrophil, and other immune cell regulation at the site of infection and inflammation [4,5]. After stimulation, platelets release cytokines, such as chemokine ligand (CXCL) 4 and 7 adhesion molecules and immune modulators (such as serotonin, and P-selectin), leading to numerous signaling events [4,5,6,7].

In this study, we aimed to analyze the CXCL4, CXCL7, serotonin, and P-selectin roles in differentiating and predicting SBI in the early phase of infection. We hypothesized that there will be differences between children referred to PED with the early symptoms of SIRS depending on the etiology of the infection (viral or bacterial).

## 2. Methods

### 2.1. Study Design and Study Population

We used data from a previous prospective single-center study conducted in the Hospital of the Lithuanian University of Health Sciences Kauno Klinikos (LSMU KK). Some additional data analysis and calculations were performed.

Children from 1 month to 5 years presenting with SIRS up to 12 h from the first onset of fever were randomly included in the study. Children who were older than 5 years, arrived at PED later than 12 h, children whose parents or legal guardians refused to participate, had psychiatric disorders or neurological disabilities, chronic diseases, immunodeficiencies, and those who received antibiotics prior to or on arrival to PED were excluded. Patients were followed until the final diagnosis and divided into three groups accordingly: viral, bacterial, or SBI (including sepsis). Nasopharyngitis, acute upper respiratory tract infection, or pharyngitis together with clear improvement without antibacterial therapy, no alterations in GBC or CRP (if repeated), and no confirmed bacterial foci (if blood, urine, or other culture was performed) were included in the viral group. Bacterial infection was confirmed if children were diagnosed with tonsillitis with a positive Strep-test or throat culture, clear signs and symptoms of adenoiditis improved with antibiotic therapy, and other not-complicated bacterial infections improved with antibiotic therapy. The SBI group included patients with complicated or more serious bacterial infections, such as bacterial pneumonia defined with clear clinical symptoms and focal infiltration on chest X-ray; pyelonephritis which was diagnosed with characteristic clinical symptoms, confirming a urine sample and positive urine culture; meningitis with representative clinics and a positive cerebrospinal fluid sample and culture; or sepsis). Sepsis was determined by clinical features (fever higher than 38 °C or lower than 36 °C, tachycardia or bradycardia, tachypnea), abnormal leucocyte counts, or more than 10% of immature neutrophil forms with or without a positive blood culture.

### 2.2. Ethical Consent

Permission to conduct the study was issued by the Kaunas Regional Biomedical Research Ethics Committee (BEC-MF-225, 23 January 2020). The study was carried out by following the principles outlined in the Declaration of Helsinki.

### 2.3. Data Collection

The following data were collected from an electronic data system: demographic data (age, gender), time of arrival, and physiological parameters, such as heart rate (HR), arterial blood pressure (ABP), blood oxygen saturation (SpO2), respiratory rate (RR), capillary refill time (CRT) and body temperature (T). Further, tachycardia (based on Advanced Pediatric Life Support guidelines), and tachypnoea (idem) were assessed according to the child’s age, and SpO2 < 94% on arrival.

### 2.4. Laboratory Measurements and Data

Next, blood was sampled for GBC and CRP analysis, and the results were included in the data analysis. Additional blood samples were collected for further protein analysis. Blood samples were handled, centrifuged, and stored according to the hospital laboratory SOPs. From the first GBC performed on arrival, white blood cells (Leu), neutrophils (Neu), platelets (PLT), mean platelet volume (MPV), platelet distribution width (PDW), platelet-large cell ratio (P-LCR), and the volume occupied by platelets in the blood (PCT) data were collected. Additionally, the platelet-to-neutrophil ratio (PNR), platelet × neutrophil-to-lymphocyte ratio (PNLR), and platelet-to-mean platelet volume ratio (PLT/MPV) were calculated.

### 2.5. Protein Analysis

In order to evaluate the CXCL4, CXCL7, serotonin, and soluble P-selectin (sP-selectin) roles in SIRS etiology (viral, bacterial, SBI, sepsis), additional blood samples were collected according to the criteria of inclusion (as previously described). After sample centrifugation, blood plasma was received. All blood plasma samples were stored at −80 °C until further use. After all samples were collected, protein analysis using an immunoassay (ELISA) was performed according to the manufacturer’s instructions (Human PF4/ CXCL4 ELISA Kit (Abcam, Cambridge, UK), NAP-2/PPBP (CXCL7) Human ELISA Kit (ThermoFisher Scientific, Frederick, MD), P-selectin (soluble) (CD62) human ELISA kit (Invitrogen, CA, USA), serotonin ELISA kit (Abcam, Cambridge, UK)). All four biomarkers were determined with the colorimetric method (wavelength 450 nm, microplate reader Multiscan Go 1.00.40 (ThermoFisher Scientific).

### 2.6. Sample Size Calculation and Statistical Analysis

The sample size was determined as follows: first, we identified around 25,000–30,000 yearly visits in the previous years. After a thorough analysis, 45% were identified as children with a fever. In addition, only 43% of the remaining cases were between 1 month and 5 years of age (our target population). Taking into account a CI of 95%, a margin of error of 5%, and considering SBI from all the feverish children within the age range, the final sample size was 100 children. Finally, 92 children were randomly selected into the study. The defined sample size was not reached as both parents, or one of them, refused to sign the consent form. In addition, after a thorough sample revision, 68 children were included in the final analysis. Others were excluded due to various reasons, such as lack of data (laboratory results, vital parameters, etc.), unclear final diagnosis, and unknown chronic conditions on arrival to PED but clarified during the hospitalization.

Data analysis was performed using Microsoft Excel and IBM SPSS Statistics version 27.0 software (SPSS Inc., Chicago, IL, USA) for Windows. The Shapiro–-Wilk test was used to determine whether the data were normally distributed. Continuous variables are expressed as mean ± standard deviation (SD) or median and interquartile range (IQR). Qualitative data are presented as counts and percentages. Our sample was divided according to previous criteria into viral, bacterial, and SBI (including sepsis) or into all bacterial (bacterial and SBI) versus viral. Continuous variables of two groups were compared by the independent samples *t*-test if the data were normally distributed, and Wilcoxon signed-rank and Mann–Whitney U tests were used to compare nonparametric data. One-way Anova was used to compare three means from three individual groups. Sensitivity and specificity were calculated for all the variables. Prognostic factors were analyzed using multivariate logistic regression analysis. Multivariate logistic regression was conducted to calculate the coefficients of biomarker combinations in predicting SBI and sepsis. The multivariate analysis results were summarized by estimating the odds ratios (OR) and the relevant 95% confidence intervals (Cl). A comparison of the diagnostic accuracy of routine inflammatory biomarkers and platelet proteins was performed using receiver operating characteristics curves (ROC) analysis. Youden’s index was used to determine the cutoff values. A *p*-value of <0.05 was considered significant.

## 3. Results

### 3.1. General Characteristics

Overall, 68 children with a median age of 2 (0–5) years were included; 37 (54.41%) were male. Viral infection (VI) was confirmed for 42 (61.8%), 10 children (14.7%) were diagnosed with bacterial infection (BI), and serious bacterial infection (SBI) was diagnosed in 16 (23.5%) children, of whom 4 children had confirmed sepsis (Table 1). We also evaluated abnormal clinical features such as tachycardia, tachypnoea, and saturation of oxygen (SpO2). The mean fever of the patients was 38.8+/−0.89 °C, 21 (31.3%) had tachypnoea, 8 (11.8%) had oxygen saturation lower than 94%, and tachycardia occurred in 33 children (48.5%) (Table 1).

### 3.2. Standard Blood Biomarkers and Derivates

All participants received GBC and CRP according to the local viral/bacterial infection diagnostics algorithm. Statistically significant differences in neutrophil and leukocyte counts were observed in bacterial infection compared to viral (*p* < 0.001) and between SBI and other groups (*p* = 0.024) (Table 2).

GBC standard platelet values PLT, MPV, PDW, P-LCR, and PCT were measured. Additionally, derivative values like PNR, PLT/MPV, and PNLR were added to the count. These values were evaluated between the viral and bacterial (SBI + other bacterial) groups. Statistically significantly higher PNLR values were noticed in the bacterial group (*p* = 0.003). Meanwhile, PNR levels were statistically significantly higher in the viral group (*p* = 0.028) (Table 2). No clear difference was observed when comparing sepsis to other causes of SIRS (Table 2). The PLT/MPV rate did not differ significantly between the groups (Table 2).

### 3.3. Platelet-Derived Markers

Platelet-derived chemokines CXCL4 and CXCL7 were compared between all bacterial infection and viral cases, but no statistically significant difference was noted (*p* = 0.348, *p* = 0.132, respectively) (Table 3). CXCL4 and CXCL7 levels in the SBI group versus others did not differ significantly. We observed statistically significantly higher CXCL7 levels in the sepsis group compared to all other cases (*p* = 0.015) (Table 3).

When investigating sP-selectin, we detected clearly higher values in the bacterial group compared to the viral group, and it was statistically significant (52.89+/−19.76 vs. 29.09+/−17.53, respectively, *p* ≤ 0.001) (Table 3). We separately compared the SBI group against all other cases, and a clear difference was detected (53.66+/−22.5 vs. 33.13+/−19.04 respectively, *p* ≤ 0.001). After we compared sepsis between all the cohorts, statistically significantly increased values were found in a sepsis group (62.25+/−17.18 vs. 35.55+/−21.08, respectively, *p* = 0.020). These data show that sP-selectin can discriminate between early viral and bacterial infections, as well as SBI from other conditions and sepsis in all other cases (Table 3).

Meanwhile, we did not find any statistically significant difference comparing serotonin levels between all the groups (Table 3).

### 3.4. Sepsis and SBI Prediction 

We hypothesized the ability of platelet activation markers to help identify sepsis according to our last findings. With the cutoff value of 31.76 pg/mL, CXCL4, as well as serotonin (data not shown), failed to discriminate sepsis from other causes (Table 4). Another platelet activation marker CXCL7 with a concentration of <95.05 pg/mL showed a sensitivity of 81.25% and specificity of 75% to distinguish sepsis compared to other causes of SIRS (AUC = 0.912, *p* = 0.006), but it could not differentiate between SBI and other causes or viral versus all bacterial (Table 4). After further analysis, sP-selectin demonstrated the power to discriminate and predict sepsis from all the other cohorts. With the cutoff value of >59.59 pg/mL and AUC of 0.847, the likelihood ratio to predict sepsis was 3.17 (*p* = 0.017) (Table 4)**.** CXCL4 and serotonin were not sensitive nor specific to predict SBI or discriminate viral versus all bacterial causes (data not shown).

The combination of CXCL7 and P-selectin with the AUC of 0.935 (*p* < 0.001) slightly increased sensitivity but specificity to predict sepsis (82.3 and 100% respectively). It was less sensitive and less specific in SBI prediction or predicting viral infection (Table 5).

Adding clinical parameters to the prediction model (tachypnoea and SpO2 <94% on arrival), we did see an improved prediction of SBI and sepsis with the AUC 0.962 and 0.973, respectively (both *p* < 0.001). Both models showed higher sensitivity and specificity in sepsis prediction (91.2% and 100% versus 96.8% and 100%, respectively).

Multivariate logistic regression was conducted to calculate the coefficients of the biomarkers’ combinations when used in predicting SBI and sepsis (Supplementary Tables S1 and S2)

## 4. Discussion

This is the first pediatric study examining the role of platelets and platelet markers in early viral versus bacterial diagnostics. Our study revealed the potential of specific platelet activation markers, such as CXCL7 and sP-selectin alone or in combination in differentiating SBI and sepsis versus other sources of infection in children presenting up to 12 h from the symptom start.

Fever is the most common reason for children’s referral to the PED [8]. Regardless of the tremendous progress in medical diagnostics, it is still very challenging to determine the cause of fever, especially at the very beginning of the infection. Lately, more new diagnostic markers have been researched to distinguish early viral versus bacterial infection (including sepsis). The existence of discriminating biomarkers as such during an infectious or non-infectious cause (e.g., trauma) could lead to better screening, diagnosis, prognosis (risk stratification), therapeutic response monitoring, and antibiotic rationalization [9,10,11,12].

Standard blood biomarkers such as leukocytes, neutrophils, and CRP have been evaluated, and they are the most commonly used biomarkers to date in differentiating viral versus bacterial infections. However, these biomarkers are not always sensitive and specific, especially in an early onset of a bacterial/viral infection. Another drawback of these biomarkers is that they cannot predict which child will develop SBI and sepsis [13]. In our study, we did observe differences in GBC, leukocyte, and neutrophils as well as CRP levels between the groups. However, in real clinical practice, it is quite complex to distinguish and relate to those biomarkers, e.g., GBC mostly can be higher in bacterial cases but still be within the pediatric norms; meanwhile, some viral pathogens can give increased CRP values [14]. As it is known, neutrophils are the most common form of granulocyte and play an important role in the innate immune system [15]. Following an infection or tissue injury, neutrophils react quickly to inflammatory cues and migrate to the inflamed/damaged area [16]. According to our findings, in a bacterial infection, higher levels of neutrophil (*p* < 0.001) and leucocyte (*p* < 0.001) counts were observed when compared to a viral infection; however, they were not exceeding the normal range for pediatric patients under 6 years of age [17].

Initially, platelets were defined as important cells in keeping hemostasis and preventing excessive bleeding. Currently, they are identified as first-line indicators in the detection and response to various pathogens [18]. Several studies have shown platelet activation under specific viral stimuli [7,19,20,21]. Moreover, platelet-neutrophil aggregates participate in the process of phagocytosis [22,23]. To analyze platelet function, first, we tested some specific platelet markers from GBC and derivatives (as described in the Methods). It has been shown that platelets do change in size and volume when stimulated [24,25]. We found no significant changes in MPV, PDW, P-LCR, or other platelet markers between the study groups. Only PNR and PNLR were significantly different between the viral and other groups and two times higher in SBI compared to other infections. However, in clinical practice, those derivative markers are quite difficult to evaluate as there are no confirmed values in a pediatric population. Thus, more studies should be performed to identify normal values in healthy children and further studied in different clinical conditions (e.g., chronic inflammation, trauma, and acute infection).

Under activation, platelets do increase in size as they produce specific granules packed with various active proteins [26,27,28]. Platelets express many proteins from their granules, which play an important part in inflammation and immune responses. In our study, we chose to evaluate four platelet proteins: sP-selectin, CXCL4, CXCL7, and serotonin.

P-selectin is capable of adhering to P-selectin glycoprotein ligand-1 (PSGL-1) on endothelial cells and neutrophils. It acts as an enhancer for platelet–neutrophil interactions in conjunction with phagocytosis. Zonneveld et al. found that sP-selectin levels were increased in sepsis patients compared to healthy controls [29]. Vassilou et al. demonstrated that in an intensive care setting, elevated sP-selectin levels distinguish between septic and non-septic patients [30]. A study by Schrijver et al. showed that sP-selectin levels are significantly related to infected patients who did not have an infection at the time of admission [31]. However, all the data are present in the adult population and in a late phase of infection. Our goal was to identify if this protein could distinguish between the early signs of infection and contribute to early SBI and sepsis recognition. In our study, sP-selectin values could discriminate between viral versus bacterial infections, and they could significantly distinguish SBI. Moreover, with the cutoff value of 59.59 pg/mL, the likelihood ratio (LR) to predict sepsis was 3.17 (AUC 0.847, *p* = 0.017). Thus, sP-selectin could be a promising marker to identify early SBI and sepsis patients.

Platelet α-granules have been confirmed to contain CXCL4 and CXCL7, which are released during the inflammatory processes [20]. These chemokines activate leukocytes and help them reach the inflammation region and induce leukocyte differentiation [32,33]. They also have an anti-inflammatory effect against bacterial and viral pathogens [34,35]. However, we were not able to find any previous studies evaluating CXCL4 and CXCL7 concentrations and their roles in infectious pathogenesis in pediatric populations. Our results show that CXCL7 is an acute inflammatory respondent. Its concentration was higher in children diagnosed with sepsis compared to other infections (*p* = 0.015). After evaluating CXCL7 sensitivity and specificity, we observed that it has potential in early sepsis diagnosis and the ability to exclude other SIRS causes (with CXCL7 concentration being ≤95.05 pg/mL, its sensitivity and specificity were, accordingly, 81.25% and 75%, *p* = 0.006). The rise of CXCL7 concentration shows that even at an early phase (<12 h since the onset of the symptoms), platelets are activated and release chemokines necessary to attract neutrophils and form NETs, while CXCL7 is one of the main proteins attracting neutrophils to the site of inflammation [36]. These detections let us hypothesize that CXCL7 has potential in early sepsis diagnosis. On the other hand, studies evaluating CXCL4 concentrations show that it plays an important role during viral infection too [20,37]. Data from an animal study with the influenza virus infection showed that lower CXCL4 concentrations resulted in decreased neutrophil chemotaxis and in the more rapid spread of the infection [37]. Interestingly, our study results showed that CXCL4 had a tendency to be higher in other infections (bacterial, SBI) compared to the sepsis group, but it was not significant (*p* = 0.162). However, after evaluating the sensitivity and specificity of CXCL4, it turned out to be not predictive enough in sepsis diagnosis and differentiation (CXCL4 value being ≤31.76, sensitivity and specificity were, accordingly, 87.5% and 75%, *p* = 0.167). This outcome may have been influenced by our relatively small sepsis and SBI group. For this reason, CXCL4 potential in SIRS etiology differentiation must be reappraised in larger studies. Nevertheless, CXCL7 and sP-selectin together could discriminate between viral, SBI, and sepsis from all the cohorts. In addition, when adding clinical signs and symptoms (tachypnoea and SpO2 <94%), it improved sensitivity and specificity to discriminate between the sepsis and the no sepsis group as well as to predict SBI. Thus, combining both markers together and adding important clinical signs could benefit a pediatric patient to identify a serious bacterial infection at an early phase of infection (<12 h after initial signs).

## 5. Limitations

The first limitation is our study sample size. We had only four cases of sepsis. With this limitation, our practical recommendation is to include more sepsis and bacterial infection cases in future studies. The second limitation is due to our initial sampling. The viral diagnosis was made only by clinical signs and with the final diagnosis, so there could be some biases as well. For the next study, a significant improvement would be viral sampling and testing. This could identify not only viral infections but mixed–viral and bacterial infections too. Thus, it could answer some questions, such as why some children with bacterial infection did respond differently (with different changes in biomarkers) than others.

## 6. Conclusions

Our study is the first study analyzing the potential of platelet biomarkers to discriminate SBI including sepsis from etiologies of SIRS in pediatric patients. Our findings showed that sP-selectin can clearly differentiate between bacterial and viral infections and with a likelihood ratio of 3.17 could predict sepsis. Other platelet-derived chemokine CXCL7 could predict sepsis as well. The combination of both markers showed significance to discriminate between all the etiological causes of SIRS. In addition, clinical signs and symptoms improved sensitivity and specificity to differentiate viral, SBI, and sepsis from other causes of infection.

Overall, the results of this study let us make the conclusion that sP-selectin and CXCL7 may have the potential to diagnose sepsis early and sP-selectin could be used to differentiate viral/bacterial, SBI/other infections, and sepsis earlier than other standard biomarkers which are used nowadays.

## Figures and Tables

**Table 1:** General characteristics of the study.

	**Total *n* = 68 (0–5 y)**
Age <12 mo (%)	23 (33.8)
Median age (IQR)	2 (0–5)
Gender (male) (%)	37 (54.41)
Time of arrival (h) (IQR)	7 (3–10)
**Clinical signs and symptoms on presentation:**	
Mean fever +/−SD	38.8+/−0.89
Tachypnea (%)	21 (31.3)
Median RR (IQR)	29 (24–36)
SpO2 <94% (%)	8 (11.8%)
Mean SpO2 +/−SD	96.85+/−3.43
Tachycardia (%)	33 (48.5%)
Median HR (IQR)	145.5 (130.5–165)
CRT +/−SD	2.34+/−0.73
**Diagnosis group**	
Viral (%)	42 (61.8)
Bacterial (%)	10 (14.7)
SBI (%)	16 (23.5) Sepsis *n* = 4

*n*-number of participants, mo-months, h-hours, y-years, RR-respiratory rate, HR-heart rate, CRT-capillary refill time, SBI-serious bacterial infection; IQR-interquartile range, SD-standard deviation.

**Table 2:** Standard blood biomarkers and derivates.

Biomarker	Overall *n* = 68	Viral *n* = 42	All Bacterial *n* = 26	*p* Value	SBI *n* = 16	Other *n* = 52	*p* Value	Sepsis *n* = 4	Other *n* = 64	*p* Value
**Leu × 10^9^/L**	11.86 ± 6.24	**9.183 ± 3.76**	**16.18 ± 7.05**	**<0.001**	**14.92 ± 7.9**	**10.92 ± 5.37**	**0.024**	11.61 ± 10.49	11.88 ± 6.01	0.288
**Neu × 10^9^/L**	7.55 ± 5.67	**4.95 ± 3.07**	**11.76 ± 6.41**	**<0.001**	**11.01 ± 7.55**	**6.49 ± 4.54**	**0.005**	7.88 ± 9.22	7.53 ± 5.49	0.324
**CRP, mg/L (IQR)**	16.38 (1.59–16.51)	**6.01 (3.49–8.59)**	**33.09 (13.06–53.11)**	**<0.001**	**59.86 (11.26–75.05)**	**11.89 ± (4.84–11.45)**	**<0.001**	**87.84 (59.22–116.56)**	**11.92 (4.76–19.07)**	**0.001**
**PLT × 10^9^/L**	292.13 ± 96.19	276.95 ± 80.45	311.81 ± 116.31	0.054	320 ± 122.18	283.56 ± 86.29	0.187	304.3 ± 160.4	291 ± 92.76	0.498
**MPV, fL**	9.47 ± 0.74	9.57 ± 0.79	9.31 ± 0.65	0.167	9.46 ± 0.58	9.48 ± 0.79	0.931	9.88 ± 0.22	9.45 ± 0,76	0.056
**PDW, fL**	10.53 ± 1.42	10.66 ± 1.58	10.33 ± 1.10	0.380	10.51 ± 0.95	10.54 ± 1.54	0.946	11.15 ± 0.39	10.50 ± 1.45	0.070
**P-LCR, %**	20.52 ± 6.17	21.19 ± 6.67	19.43 ± 5.23	0.255	20.53 ± 4.62	20.51 ± 6.62	0.991	23.53 ± 1.42	20.33 ± 6.31	0.074
**PCT**	0.28 ± 0.09	0.27 ± 0.07	0.29 ± 0.10	0.279	0.30 ± 0.11	0.27 ± 0.08	0.125	0.25 ± 0.16	0.28 ± 0.08	0.054
**PLT/MPV (IQR)**	31.28 (23.73–36.02)	29.64 (26.72–32.55)	33.93 (28.47–39.39)	0.126	34.17 (26.85–41.48)	30.39 (27.51–33.26)	0.242	24.02 (12.89–35.17)	31.73 (28.94–34.51)	0.185
**PNR (IQR)**	69.11 (29.39–88.29)	**82.71 (63.16–102.26)**	**47.15 (21.29–73.02)**	**0.028**	60.54 (18.51–102.57)	71.75 (54.79–88.71)	0.551	67.43 (1.92–132.93)	69.22 (52.85–85.6)	0.958
**PNLR (IQR)**	1362.32 (705.14–2128.68)	**945.42 (609.24–1281.60)**	**2035.78 (1303.3–2768.27)**	**0.003**	**2161.43 (1025.87–3296.99)**	**1116.44 (796.05–1436.85)**	**0.013**	518.78	1414.98 (1043.78–1786.19)	0.247

Results are expressed as standard deviations. SBI-serious bacterial infection, *n*-number of participants, Leu-leukocytes, Neu-absolute neutrophil count, CRP-C-reactive protein, PLT-platelets, MPV-mean platelet volume, PDW-platelet distribution width, P-LCR-platelet large cell ratio, PNR-platelet-to-neutrophil ratio, PLT/MPV-platelet count and mean platelet volume ratio, PNLR-platelet count multiplied by neutrophil-lymphocyte count, PCT-platelet, mg/l-milligrams per liter, L-liter; fL-femtoliter (dm3), IQR-interquartile range; the significant level at *p* < 0.05.

**Table 3:** Platelet biomarkers values according to the etiology of the infectious process.

	Overall *n* = 68	Viral *n* = 42	All Bacterial *n* = 26	*p* Value	SBI *n* = 16	Other *n* = 52	*p* Value	Sepsis *n* = 4	Other *n* = 64	*p* Value
**CXCL4 (pg/mL)**	36.3 ± 4.8	35.52 ± 4.49	34.38 ± 5.32	0.348	34.38 ± 6.15	35.30 ± 4.38	0.505	31.81 ± 5.54	35.29 ± 4.74	0.162
**CXCL7 (pg/mL)**	84.6 ± 10.9	82.20 ± 10.34	86.31 ± 11.50	0.132	88.1 ± 11.36	82.44 ± 10.51	0.069	**96.5 ± 4.87**	**82.98 ± 10.7**	**0.015**
**sP-selectin (pg/mL)**	34.7 ± 21.6	**29.09 ± 17.53**	**52.89 ± 19.76**	**<0.001**	**53.66 ± 22.5**	**33.13 ± 19.04**	**<0.001**	**62.25 ± 17.18**	**36.55 ± 21.08**	**0.020**
**Serotonin (pg/mL)**	16.6 ± 4.1	16.34 ± 4.22	16.90 ± 4.02	0.603	17,04 ± 3.99	16.4 ± 4.19	0.590	15.28 ± 4.6	16.64 ± 4.12	0.527

Results are expressed as standard deviations. *n*-number of participants, CXCL4-chemokine ligand 4; CXCL7-chemokine ligand 7; VI-viral infection; BI-bacterial infection; SBI-severe bacterial infection; pg/mL-picograms per milliliter; the significant level at *p* < 0.05.

**Table 4:** CXCL7, and sP-selectin AUC, likelihood ratio, sensitivity, and specificity values.

Marker	Compared Groups	Cutoff Value pg/mL	Youden’s Index	AUC (CI 95%)	Likelihood Ratio	Sensitivity %	Specificity %	*p* Value
**CXCL7**	Sepsis (*n* = 4) vs. other infections (*n* = 64)	95.05	0.560	0.912 (0.824–1.000)	3.25	81.25	75	0.006
	SBI (*n* = 16) vs. other (*n* = 52)	81.20	0.197	0.644 (0.493–0.815)	2.05	38.46	81.25	0.080
	VI (*n* = 42) vs. all bacterial etiology infections (*n* = 26)	91.69	0.214	0.642 (0.467–0.758)	1.75	50	71.43	0.050
**sP-selectin**	Sepsis (*n* = 4) vs. No sepsis (*n* = 64)	59.59	0.544	0.847 (0.660–1.001)	3.17	79.37	75	0.017
	SBI (*n* = 16) vs. other (*n* = 52)	52.98	0.465	0.754 (0.603–0.905)	2.33	84.0	62.5	0.001
	VI (*n* = 42) vs. all bacterial etiology infections (*n* = 26)	38.06	0.550	0.807 (0.695–0.919)	3.78	75.6	80	<0.001

CXCL7-chemokine ligand7, AUC-area under the curve, *n*-number of participants, VI-viral infection; BI-bacterial infection, SBI-severe bacterial infection; pg/mL-picograms per milliliter.

**Table 5:** Prediction of viral infection, SBI, and sepsis of a combination of CXCL7, and sP-selectin or CXCL7, sP-selectin, tachypnoea, and SpO2.

Marker (Cutoff Value pg/mL)	Compared Groups	Cutoff Value	Youden’s Index	AUC (CI 95%)	Sensitivity %	Specificity %	*p* Value
**CXCL7 + sP-selectin**	**VI vs. bacterial etiology of infection**	0.003	0.339	0.728 (0.598–0.858)	65.9	68	0.001
**CXCL7 + sP-selectin**	**SBI vs. other**	0.050	0.402	0.738 (0.586–0.889)	85	56.2	0.002
**CXCL7 + sP-selectin**	**Sepsis vs. no sepsis**	0.054	0.823	0.935 (0.855–1.016)	82.3	100	<0.001
**CXCL7 + sP-selectin + tachypnoea**	**Sepsis vs. no sepsis**	0.130	0.919	0.962 (0.909–1.016)	91.8	100	<0.001
**CXCL7 + sP-selectin + tachypnoea + SpO2 < 94%**	**SBI vs. other**	0.289	0.613	0.815 (0.671–0.985)	88	73.3	<0.001
**CXCL7 + sP-selectin + tachypnoea + SpO2 < 94%**	**Sepsis vs. no sepsis**	0.227	0.968	0.973 (0.934–1.012)	96.8	100	<0.001

CXCL7-chemokine ligand 7, VI-viral, SBI-serious bacterial infection, AUC-area under the curve.

## Data Availability

Not applicable.

## Supplementary Materials

The following supporting information can be downloaded at: https://www.mdpi.com/article/10.3390/jcm11216475/s1, Table S1: Univariant logistic regression analysis to predict SBI and sepsis; Table S2: Multivariant logistic regression analysis to predict SBI and sepsis.
