# Supplementary material for: Platelet-Derived Biomarkers: Potential Role in Early Pediatric Serious Bacterial Infection and Sepsis Diagnostics
Source: J Clin Med. 2022 Oct 31;11(21):6475. doi: 10.3390/jcm11216475 (PMC9658833; doi:10.3390/jcm11216475)
Supplement: Supplementary file 1 [file jcm-11-06475-s001.zip › jcm-1915939-supplementary.pdf]

Supplementary Mterials

Table S1. Univariant logistic regression analysis to predict SBI and sepsis.

		Coeff. B	SE	WALD	*p*-Value	OR	CI 95%
SBI vs. others	CXCL7	0.055	0.031	3.180	0.075	1.056	0.995–1.122
	constant	−5.861	2.689	4.751	0.029	0.003	
	sP-selectin	0.050	0.016	9.358	0.002	1.051	1.018–1.085
	constant	−3.307	0.845	15.324	0.000	0.037	
	CRB	0.052	0.019	7.407	0.006	1.053	1.015–1.093
	constant	−2.043	0.436	21.990	0.000	0.130	
Sepsis vs. others	CXCL7	0.233	0.111	4.411	0.036	1.263	1.016–1.569
	constant	−24.075	10.612	5.147	0.023	0.000	
	sP-selectin	0.067	0.034	3.941	0.047	1.069	1.016–1.569
	constant	−6.119	2.110	8.411	0.004	0.002	
	CRB	0.032	0.014	4.995	0.025	1.033	1.004–1.062
	constant	−3.786	0.808	21.964	0.000	0.023	

SE—standard error, OR—odds ratio, CI—confidence interval, CXCL7—chemokine ligand 7, CRB-C-reactive protein, SBI-serious bacterial infection.

Table S2. Multivariant logistic regression analysis to predict SBI and sepsis.

		Coeff. B	SE	WALD	*p*-Value	OR	CI 95%
SBI vs. other	CXCL7	0.044	0.031	2.039	0.153	1.045	0.984–1.110
	sP-selectin	0.049	0.017	8.275	0.004	1.050	1.016–1.085
	constant	−7.002	2.795	6.276	0.012	0.001	
	CXCL7	0.032	0.032	0.956	0.328	1.032	0.969–1.100
	sP-selectin	0.041	0.018	5.286	0.021	1.042	1.006–1.080
	CRB	0.039	0.020	3.814	0.051	1.040	1.000–1.081
	constant	−6.345	2.945	4.642	0.031	0.002	
sepsis vs. other	CXCL7	0.218	0.120	3.315	0.069	1.243	0.983–1.572
	sP-selectin	0.067	0.039	2.991	0.084	1.070	0.991–1.154
	constant	−26.180	12.040	4.728	0.030	0.000	
	CXCL7	0.256	0.176	2.112	0.146	1.292	0.915–1.825
	sP-selectin	0.145	0.096	2.282	0.131	1.156	0.958–1.395
	CRB	0.032	0.017	3.662	0.056	1.033	0.999–1.067
	constant	−36.362	21.204	2.941	0.086	0.000	

SE—standard error, OR—odds ratio, CI—confidence interval, CXCL7—chemokine ligand 7, CRB-C-reactive protein, SBI-serious bacterial infection.
